# Novel natural withanolides induce apoptosis and inhibit migration of neuroblastoma cells through down regulation of N-myc and suppression of Akt/mTOR/NF-κB activation

**DOI:** 10.18632/oncotarget.24429

**Published:** 2018-02-07

**Authors:** Chitra Subramanian, Patrick T. Grogan, Valerie P. Opipari, Barbara N. Timmermann, Mark S. Cohen

**Affiliations:** ^1^ Department of Surgery, University of Michigan, Ann Arbor, MI, USA; ^2^ Department of Internal Medicine, University of Wisconsin, Madison, WI, USA; ^3^ Department of Pediatrics, University of Michigan, Ann Arbor, MI, USA; ^4^ Department of Medicinal Chemistry, University of Kansas, Lawrence, KS, USA; ^5^ Department of Pharmacology, University of Michigan, Ann Arbor, MI, USA

**Keywords:** neuroblastoma, withanolides, N-myc, Akt/mToR/NFKB, apoptosis

## Abstract

Despite recent advances in intensive chemotherapy treatments, long-term success is achieved in less than 30% of children with high-risk neuroblastoma (NB). Key regulatory pathways including the PI3K/Akt, mTOR and NF-κB are implicated in the pathogenesis of NB. Although drugs targeting these individual pathways are in clinical trials, they are not effective due to the activation of compensatory mechanisms. We have previously reported that natural novel withanolides from *Physalis longifolia* can potently inhibit these key regulatory pathways simultaneously. In the present study, we examined the efficacy and mechanisms through which novel withanolides and their acetate derivatives (WGA-TA and WGB-DA) from *P.longifolia* kill NB cells. The results from the study demonstrated that our novel acetate derivatives are highly effective in inhibiting the proliferation, shifting the cell cycle and inducing apoptosis in a dose dependent manner. Analysis of oncogenic pathway proteins targeted by withanolides indicated induction of heat shock response due to oxidative stress. Dose dependent decrease in clients of HSP90 chaperone function due to suppression of Akt, mTOR, and NF-κB pathways led to decrease in the expressions of target genes such as cyclin D1, N-myc and Survivin. Additionally, there was a dose dependent attenuation of the migration and invasion of NB cells. Furthermore, the lead compound WGA-TA showed significant reduction in tumor growth of NB xenografts. Taken together, these results suggest that withanolides are an effective therapeutic option against NBs.

## INTRODUCTION

Neuroblastoma (NB) is an aggressive neural crest derived pediatric malignancy accounting for 8–10% of all pediatric cancers and is often refractory to conventional therapies [1, 2]. NB tumors exhibit clinical and molecular heterogeneity ranging from spontaneous regression in children less than 1 year to metastatic disease in older children complicating standardized treatment regimens. Although patients with low risk respond well to treatment, majority of the NB patients are detected with malignant metastatic spread and 40–50% of these high-risk patients have N-myc oncogene amplification, which is one of the key prognostic factor of poor outcome in advanced stage NB. Despite intense multimodal therapy and improvements in understanding the molecular and genetic basis of NB, the therapeutic outcome of high risk children have a dismal long-term survival rate of only 18–30% either due to recurrence after treatment or due to the development of resistance to conventional therapy [3]. Therefore, new treatment alternatives are needed to improve the poor outcome of advanced stage NB patients.

Current treatment options for aggressive NB involves a coordinated sequence of multimodal treatment options such as induction chemotherapy, surgical resection of the primary tumor, high-dose myeloablative chemotherapy with autologous stem-cell transplantation (SCT), radiation, immunotherapy using anti-GD2 antibody and differentiation therapy using 13-cis retinoic acid (CRA) for targeting the residual tumor [4]. In addition to the standard treatment options, targeted therapies using inhibitors of molecules implicated in the pathogenesis of NB such as anaplastic lymphoma kinase (ALK), tropomyosin receptor kinase (TRK), the phosphatidylinositol 3′-kinase (PI3K)/Akt/mammalian target of rapamycin (mTOR) and the insulin-like growth factor 1 receptor (IGF1R), histone deacetylases (HDAC) and others are in phase I/II clinical trials either alone or in combination [5–10] to improve the outcome of high-risk NB patients. Despite advances in the development of targeted therapies and initial encouraging results, many patients later develop resistance to the targeted drug and relapse due to the activation of alternate pathways. Therefore, development of drugs targeting multiple pathways implicated in the pathogenesis of NB and has lower toxicity profiles are urgently needed.

Recently, natural compounds are gaining importance as anti-cancer therapeutics due to their non-toxic, anti-carcinogenic activity and approximately 50% of FDA approved drugs are either natural products or their derivatives [11, 12]. As part of our investigation to develop anti-cancer drug leads from the plant diversity of the USA, our group has been developing natural and semi-synthetic withanolides that are 28-carbon steroidal lactones [13] found in *Solanaceae* family of plants as potential anti-cancer chemotherapeutic agents for several cancers including brain, head and neck, thyroid, breast, adrenal and other tumors [14–21]. Withanolides have thiol reactivity and have shown promising anti-tumor efficacy through modulation of many cellular pathways including the PI3K/Akt/mTOR, nuclear factor-κB (NF-κB) and others [14, 18, 22–26] that are implicated in the pathogenesis of NB. They exert their anti-tumor efficacy through a mechanism of an oxidative stress response from metabolism of the epoxide in the B-ring and through their direct inhibition of HSP90/Cdc37 chaperone activity [27–31]. In addition, withanolides have a large therapeutic index and selectivity for cancer cells. Hence, they are not plagued by the resistance mechanisms that effect mono-targeted therapeutics. Therefore, they present novel potent anti-cancer therapeutics for children with NB. The main goal of the present study is to investigate the *in vitro* efficacy and mechanism of action of novel unmodified withaferin A (WA) and withalongolide A (WGA) as well as the semi-synthetic withanolides from the *Physalis* plant, namely withalongolide A 4, 19, 27-triacetate (WGA-TA) and Withalongolide B 4, 19 diacetate (WGB-DA) from *Physalis longifolia* that have shown potent anti-tumor efficacy in multiple cancer models during structure-activity relationship analysis [28].

## RESULTS

### Withanolides are cytotoxic to NB cells

**Figure 1:**
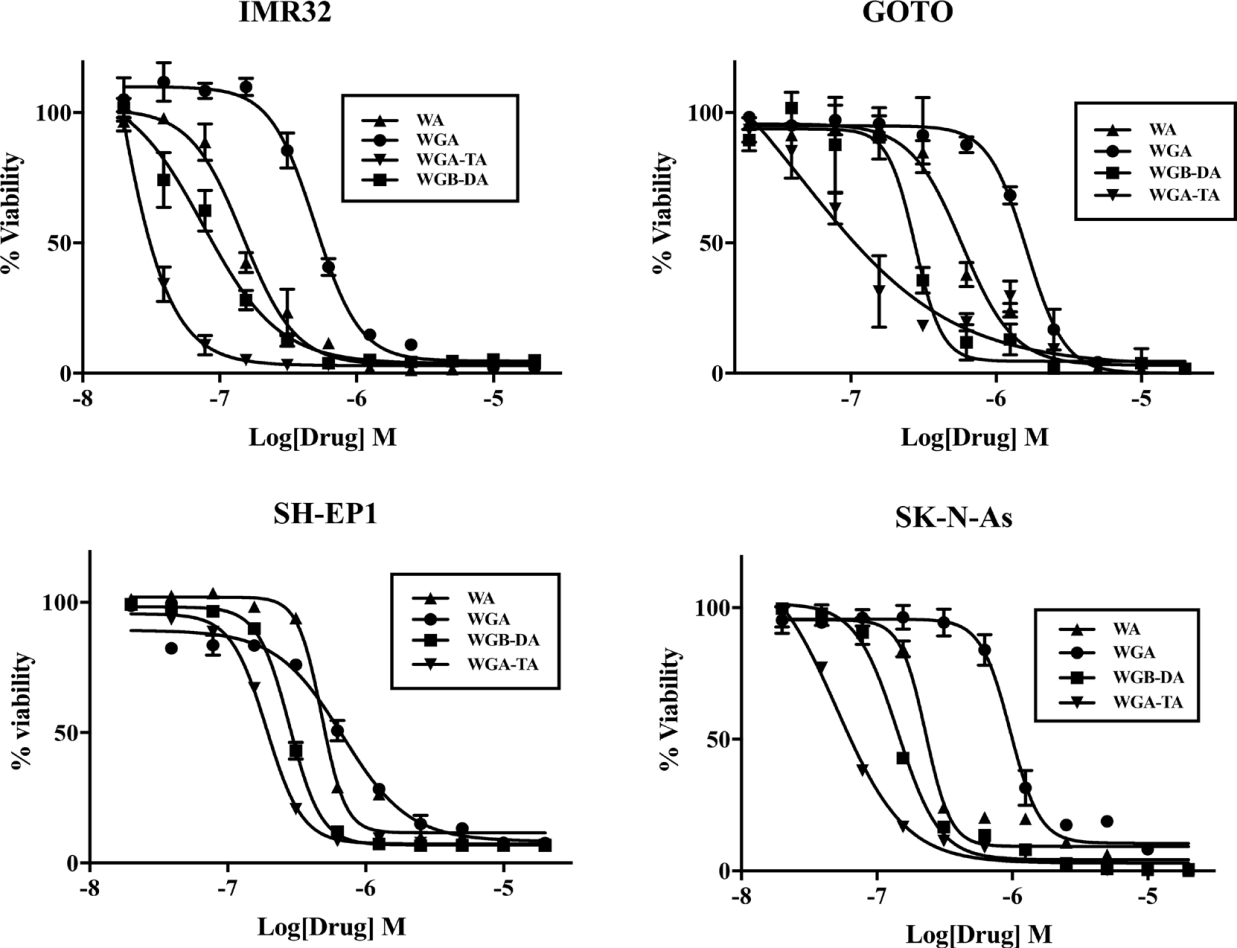
Treatment of NB cells with novel withanolides has anti-proliferative effect Cell viability was determined by MTS assay after treating NB cell lines IMR 32, GOTO, SH-EP1 and SK-N-As with varying concentrations of withanolides WA, WGB-DA or WGA-TA or solvent DMSO control for 72 h. Data presented are mean from triplicate value and error bars indicate standard error. The values are presented as percentage of control DMSO treatment.

The proliferation of four different NB cell lines after 24 h or 72 h treatment with varying concentrations of withanolides (WA, WGA, WGA-TA or WGB-DA) was evaluated using MTS cell viability assay and the IC50 values for each compound in NB cells were calculated using GraphPad Prism (Table 1A and 1B; Figure 1). Time dependent changes in IC_50_ was seen for all the cell lines tested as seen from changes in IC50 values from low μM at 24 h to low nM values. Of all the compounds tested higher efficacy for the acetate derivative compared to the parent compound were seen in all the NB cell lines tested both at 24 h and 72 h. The order of potency of withanolides tested were WGA-TA>WGB-DA>WA>WGA, indicating that the introduction of acetyl group significantly improves the potency of the parent compound. In addition fold selectivity for the most potent compound was 15–51 fold higher in NB cells compared to normal fibroblast cells (data not shown). Two human NB cell lines (IMR 32 and GOTO) and the withanolides WA, WGA-TA, and WGB-DA were used in all the subsequent mechanistic studies.

Table 1Half-maximal inhibitory concentration (IC50) values for NB cells after withanolides treatment

**Table:** A

24 h	WA	WGA	WGB-DA	WGA-TA
**IMR32**	2.09 ± 1.05	2.80 ± 0.72	1.19 ± 0.37	0.88 ± 0.23
**GOTO**	3.10 ± 1.57	4.30 ± 0.81	1.36 ± 0.47	0.89 ± 0.13
**SH-EP1**	3.42 ± 0.41	4.20 ± 0.91	1.16 ± 0.17	0.72 ± 0.23
**SK-N-As**	3.09 ± 1.16	2.98 ± 0.66	1.60 ± 0.19	0.52 ± 0.10

**Table:** B

72 h	WA	WGA	WGB-DA	WGA-TA
**IMR32**	0.30 ± 0.02	0.50 ± 0.04	0.08 ± 0.03	0.02 ± 0.02
**GOTO**	0.58 ± 0.13	1.62 ± 0.03	0.27 ± 0.06	0.04 ± 0.08
**SH-EP1**	0.51 ± 0.05	0.68 ± 0.09	0.28 ± 0.01	0.19 ± 0.01
**SK-N-As**	0.23 ± 0.03	0.97 ± 0.09	0.14 ± 0.01	0.05 ± 0.01

The NB cells were treated with varying concentrations of WA, WGA, WGA-TA or WGB-DA for 24 h (**A**) and 72 h (**B**). MTS assay was used to determine viability and the values are presented as IC_50_ values in μM ± 95% CI.

### Cell cycle distribution of NB cells is shifted after treatment with withanolides

**Figure 2:**
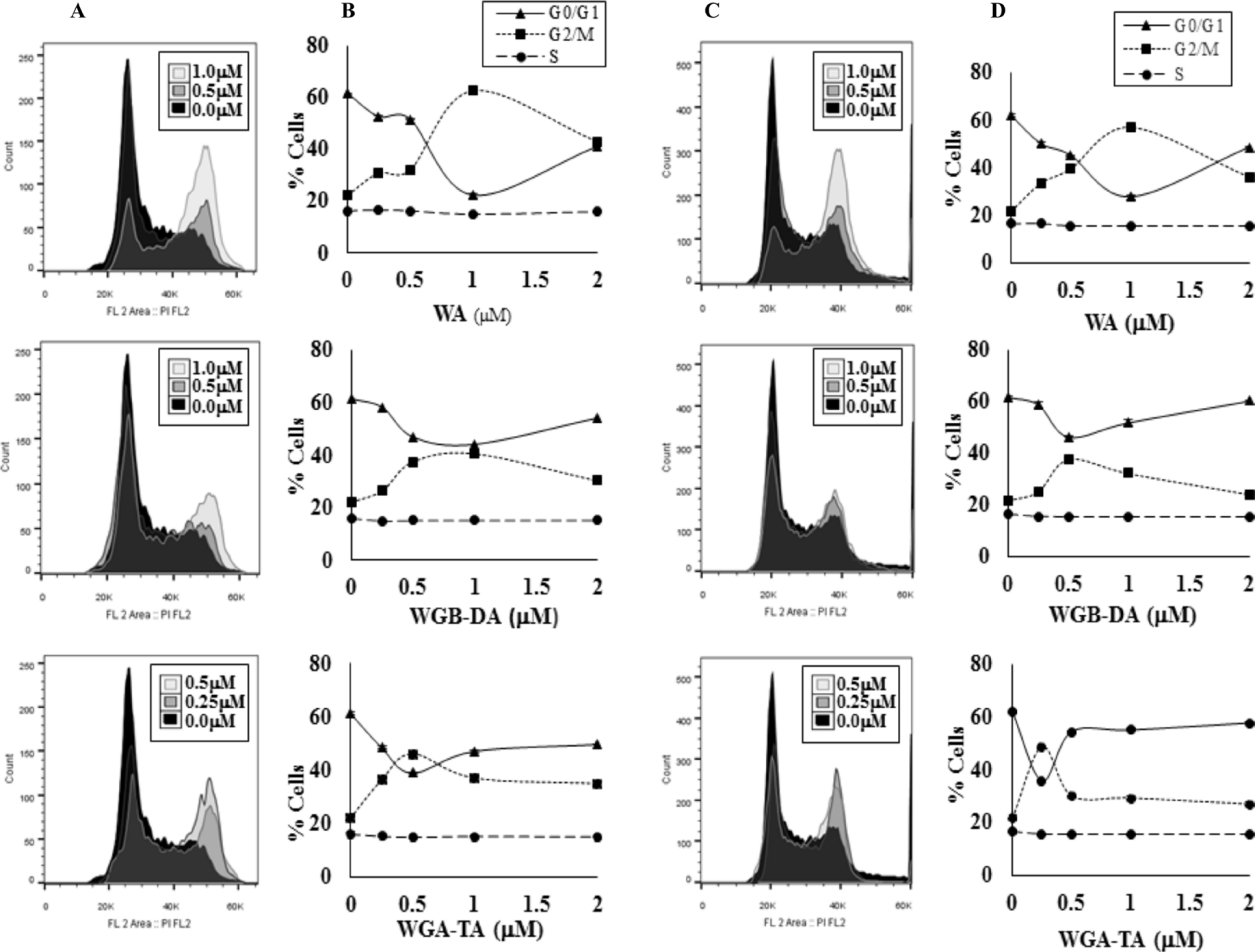
Withanolides regulate the cell cycle effect of NB cells The NB cells were stained with propidium iodide (PI) after treatment with varying concentrations of three different withanolides, WA, WGA-TA or WGB-DA, for 24 h and the cell cycle distribution was measured by flow cytometry (**A**–**B**) is IMR 32 cells and (**C**–**D**) is GOTO cells. The values of mean of three independent observations are presented.

As MTS assay absorbance measurement can be multifactorial, we assessed the distribution of cells in different phases of the cell cycle by flow cytometry after treatment with each withanolides for 24 h. Withanolide treatment of two NB cell lines IMR 32 and GOTO shifted the cells from G0/G1 phase to G2/M phase starting at 250 nM in a dose-dependent manner with minimal changes in S-phase (Figure 2A–2D). Maximum shift to G2/M phase above the basal level was observed for both NB cell lines at 1 μM, 0.5–1 μM and 0.25–0.5 μM for WA, WGB-DA and WGA-TA respectively. In IMR 32 cells the percentage of cells in G2/M increases to 62.43%, 46.07% and 40.47% after treatment with 1 μM WA, 1 μM WGB-DA and 0.5 μM WGA-TA respectively compared to 22.4% for the control untreated cells. Furthermore, in GOTO cells the percentage of G2/M increased to 56.9%, 37.93% and 48.63% after treatment with 1 μM WA, 0.5 μM WGB-DA and 0.5 μM WGA-TA respectively compared to 21.63% for the control untreated cells. In both cases, there were highly significant *p* values of 0.001. At higher drug concentrations (2–4 μM) where increase in apoptosis and cell debris was observed in the sub G0 phase, the percentage of cells in G2/M levels decreased with increases in G0/G1 levels for both the NB cell lines.

### Withanolides induce apoptosis through caspase activation and PARP cleavage in NB cells

**Figure 3:**
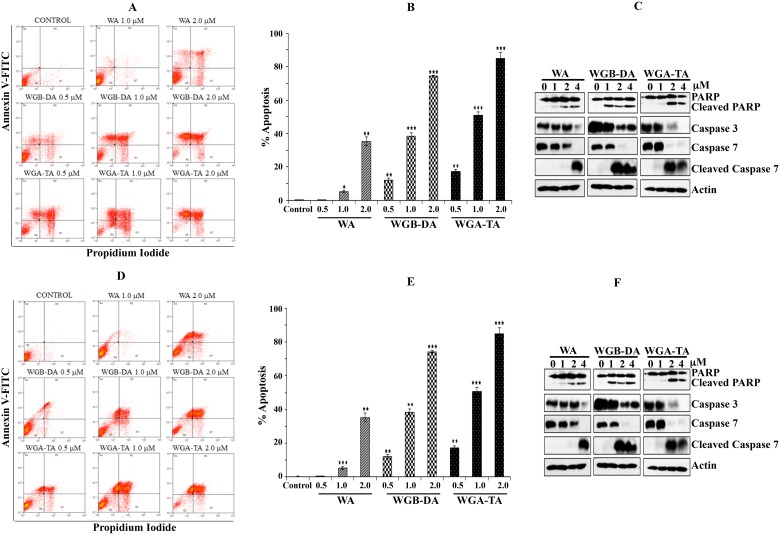
Induction of apoptosis after novel withanolides treatment in NB cells (**A** and **D**) IMR 32 and GOTO cells were stained with PI and annexin V-FITC and then analyzed by flow cytometry after treatment with increasing amounts of each withanolides. (**B** and **E**) Graphic representation of induction of apoptosis and necrosis after treatment of IMR 32 and GOTO cells with increasing concentrations of withanolides. Mean ± standard deviation results from three independent experiments are shown. (**C** and **F**) Western blot analysis of cleavage of PARP and caspases 3 and 7 after treatment of NB cells, IMR 32 and GOTO with novel withanolides for 24 h confirms induction of apoptosis. As a loading control actin was used.

To explore whether the growth suppression mechanism of NB cells observed following exposure to withanolides (WA, WGA-TA or WGB-DA) is due to apoptotic or necrotic mechanism of cell death, annexin V-FITC/PI dual staining on flow cytometry was performed. The treatment of NB cell lines GOTO and IMR 32 with increasing concentrations of each withanolide ranging from 0.5 μM–2 μM for 24 h resulted in increase in FITC-Annexin V / PI dual staining in a dose dependent manner. This result in both cell lines indicates enhanced apoptotic death with increasing drug dosage. From the basal levels of 3.6%, the number of dead cells (necrotic + late and early apoptotic) increased in a dose dependent manner to 35.5%, 90.6% and 95.2% for WA, WGB-DA and WGA-TA respectively with significant *p* values of < 0.001 at the highest concentration of 2 μM tested for IMR 32 cells (Figure 3A and 3B), with increased necrotic cells seen at low concentrations for WGA-TA. In the case of GOTO cells also the percentage of dead cells increased in a dose dependent manner from a basal level of 2.66% to 44.49%, 74.28% and 89.09% for WA, WGB-DA and WGA-TA respectively at 2 μM (Figure 3D and 3E) with a *p* value of < 0.001 for the acetate derivatives and a *p* value of 0.01 for the parent compound. Induction of apoptosis was further confirmed by examining the cleavage and/or total level of effector procaspases 3 and 7, and the downstream poly (ADP-ribose) polymerase (PARP) after treatment of both IMR 32 and GOTO with each withanolide for 24 h by western blot analysis. Treatment with increasing concentrations of withanolide resulted in progressively decreased levels of procaspases and poly ADP ribose polymerase (PARP) (Figure 3C and 3F). Cleavage of caspases 3 and 7, and PARP was observed starting at 1 μM for IMR 32 cells whereas for GOTO cells cleavage of caspases started at 2 μM although PARP cleavage occurred starting from 1 μM. Taken together the above results from the viability assay and flow cytometry analysis for cell cycle and apoptosis, indicated that the introduction of acetate derivative to the parent compound increases the potency of the drug.

### Withanolides treatment of NB cells modulates Akt/mTOR/MAPK pathway proteins

**Figure 4:**
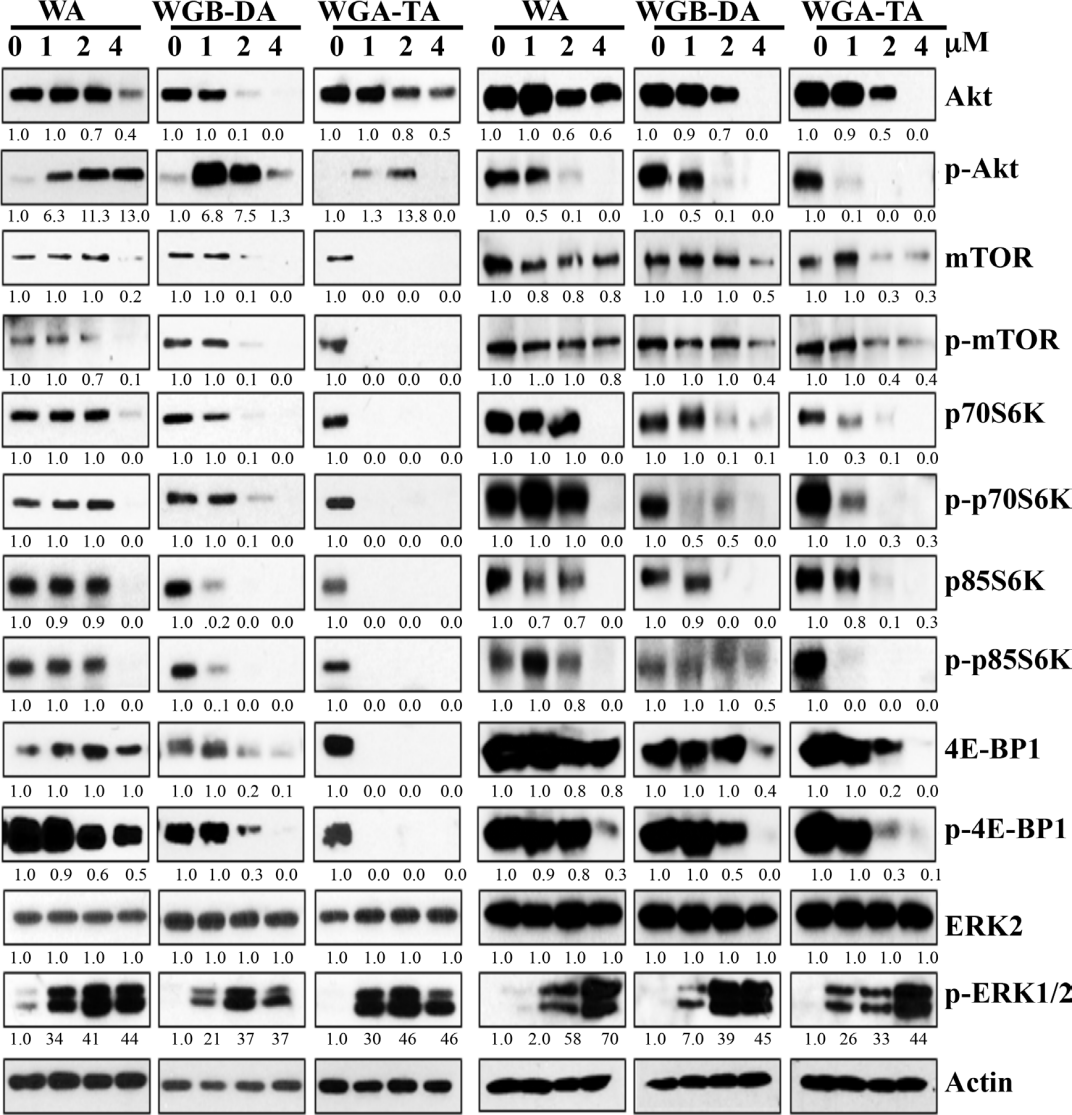
Treatment of IMR 32 (Left) and GOTO (Right) cells with withanolides modulate the proteins involved in Akt/mTOR/MAPK pathway Following treatment of the NB cells with increasing concentrations of each withanolide for 24 h, expression levels of proteins from the Akt/mTOR/MAPK pathway was measured by immunoblot analysis using appropriate primary antibodies. Results indicate modulation of proteins with increasing concentrations of withanolides. Actin was used as a loading control.

To elucidate the mechanism through which withanolides induce apoptosis and suppress the viability of NB cells, we investigated the modulation of key regulatory proteins involved in the proliferation of NB cells by western blot analysis. Since, N-myc plays a primary role in the pathogenesis and aggressiveness of NB and PI3K/Akt pathway is known to stabilize N-myc protein, we first investigated the Akt/mTOR pathway proteins after treatment of both IMR 32 and GOTO cells with increasing concentrations (1–4 μM) of each withanolide (Figure 4). At 24 h post treatment the levels of Akt decreased from 27% at 1 μM to complete inhibition by 4 μM for acetylated WGA-TA and from 8% (1 μM) to 85% (4 μM) for WA (non-acetylated, *p* < 0.01). Changes in the levels of mTOR, pmTOR and its downstream targets, p70-S6 kinase and p-4EBP1, showed 9% inhibition at 1 μM for WA to complete knockdown at 1μM for WGA-TA in both NB cell lines. These results further confirmed the higher efficacy of the acetate derivatives and indicated that the withanolide treatment of NB cells suppressed the activation of Akt/mTOR pathway proteins as well the down-stream effectors of mTOR pathway involved in translation and protein synthesis. Since MAPK and Akt pathways are compensatory pathways we next assessed the expression levels of p-ERK and ERK (Figure 4). The results indicated no variation in the expression of total ERK, whereas the level of p-ERK increased with increasing concentration of withanolides indicating activation of the compensatory MAPK pathway.

### Treatment with withanolides induces oxidative stress as well as heat shock response and pretreatment with thiol antioxident NAC blocks ROS and apoptosis of NB cells

**Figure 5:**
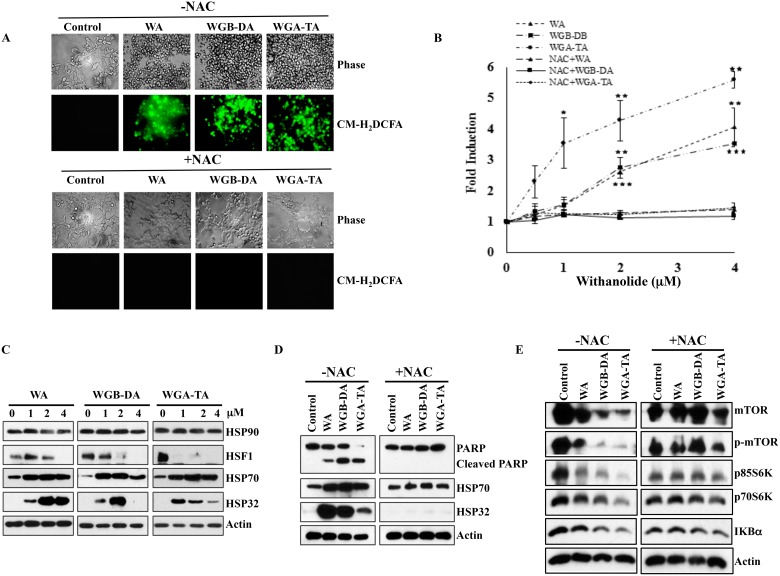
Withanolides induce cellular stress response in NB cells IMR 32 cells were preloaded with ROS indicator CM-H_2_DCFDA prior to withanolides exposure and alteration in oxidation potential was measured. (**A** and **B**) Fluorescent intensity measurement revelaed the induction of ROS after 4 h of treatment in a dose dependent manner and complete blocking of this effect by 5 mM NAC co-treatment. Immunoblot analysis of the stress response, heat shock proteins demonstrated increased expression levels of HSP32 and HSP70, and decreased expression of HSF1 after 24 h of treatment with increasing concentrations of withanolides. When the cells were pre-treated with NAC, cleavage of PARP that is an indicator of apoptosis was blocked and the increase in the expression levels of the heat shock proteins HSP32 and HSP70 as well as decrease in expression levels of mTOR pathway proteins and Iκ-Bα are blocked after NAC treatment, indicating attenuation of the anti-proliferative properties of withanolides by NAC (**C**–**E**).

Recent studies by our group and others have demonstrated that WA targets proteins involved in multiple cellular pathways through an oxidative response from the metabolism of the epoxide in the B-ring [18, 32, 33]. To determine whether withanolides induces oxidative stress in NB cells or not, we investigated induction of oxidative stress using CM-H_2_DCFDA staining method after treatment of NB cells with varying concentrations of WA and our novel acetate derivatives (WGB-DA and WGA-TA). Treatment of IMR 32 cells with 0.5–4 μM withanolides resulted in rapid and transient increase in mean DCF fluorescence peaking at 4 h that declined at 7 h. The ROS production increased in a dose dependent manner to 4.06, 3.5 and 5.6 fold for WA, WGB-DA and WGA-TA at the highest concentration of 4 μM tested with significant *p* values of 0.001, 0.002 and 0.005 respectively, compared to the control group (Figure 5A). When NB cells were treated with 5 mM NAC, which is a thiol antioxidant and ROS scavenger along with withanolides, generation of ROS was completely abrogated (Figure 5A and 5B) and returned to near basal levels with significant *p*-values of less than 0.001.

Withaferin A has been suggested to exert its anti-cancer properties through direct inhibition of HSP90/Cdc37 chaperone activity, induction of heat shock response and apoptosis following oxidative response in many cancer models [14, 18, 34]. In addition, heat shock protein HSP90 inhibition is known to induce HSP70. Furthermore, the HSP90/HSP70 chaperone machinery is known to induce degradation of HSP90 client proteins through proteasome-dependent pathway [34, 35]. Since we observed degradation HSP90 client proteins and oxidative stress, we next determined whether withanolide treatment of NB cells induced heat shock response. Consistent with previous findings, withanolide treatment of NB cells also resulted in modulation of heat shock proteins, which was significantly reduced after treatment with NAC. Even though total levels of HSP90 did not change the levels of HSP70 increased considerably starting at 1 μM withanolides for 24 h treatment, whereas levels of HSF 1 decreased in a dose dependent manner indicating inhibition of HSP90 (Figure 5C). The heat shock protein HSP32 expression increased with increasing concentrations of WA, whereas in the case of acetate derivatives the levels of HSP32 increased initially at low concentrations of 1 and 2 μM drug treatments and decreased at a higher concentration of 4 μM due to apoptosis (Figure 5C). Pretreatment of NB cells with NAC completely blocked the heat shock response, induction of apoptosis as well as down regulation of mTOR, p-mTOR, p70/85S6K, p-p70/85S6K and Iκ-Bα (Figure 5D and 5E)

### Withanolides suppress activation of NF-κB, down regulate the expression of NF-κB target genes cyclinD1, survivin and N-myc as well as prevent migration and invasion of NB cells

**Figure 6:**
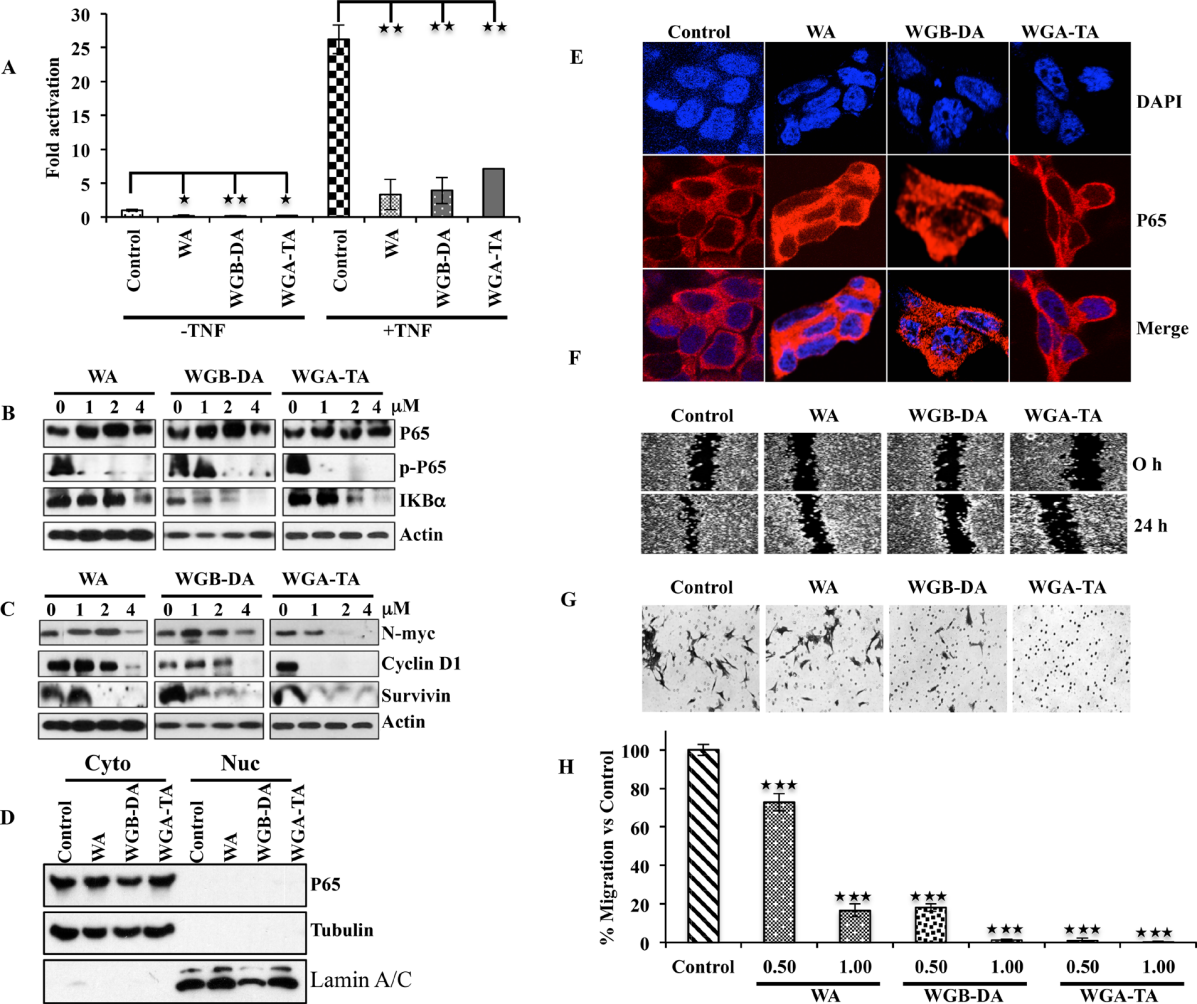
Down regulation of the NF-κB activity after withanolide treatment leads to down regulation of p-p65 (ser536), targets N-myc and prevents migration as well as invasion of NB cells (**A**) NB cell line IMR 32 was transfected with 0.5 mg of NF-kB reporter along with 50 ng of renilla luciferase by lipofectamine 2000. 24 h post transfection, the cells either were treated with 2 μM withanolides (WA, WGB-DA or WGA-TA) for 3 h before treatment with TNF-α for 16 h for TNF-α mediated NF-κB activity and left untreated for uninduced NF-κB activity. The luciferase activity was measured 24 h post addition of withanolides using Promega dual luciferase assay kit. (**B**) Western blot analysis of p-p65 (ser536) and Iκ-Bα demonstrating degradation of Iκ-Bα and down regulation of phospho-p65 (ser 536) whereas total levels of p65 remains unchanged. (**C**) Down regulation of NF-κB target genes such as N-myc, cyclin D1 and survivin in a dose dependent manner after 24 h treatment of IMR 32 cells with withanolides. (**D** and **E**). Imuuno fluorescence analysis of p65 after withanolides treatment evaluated by confocal microscopy indicated cytosolic presence of p65. Absence of nuclear staining of p65 showed inhibition of NF-κB due to prevention of nuclear localization of p65. DAPI was used for staining nuclei. This was further confirmed by the cytosolic and nuclear fractionation immunoblots. The same blot was stripped and reprobed for tubulin and lamin A/C after immunoblotting for p65 to verify the integrity of the cytosolic and nuclear fractions. (**F**–**H**). The acetate derivatives effectively block migration and invasion of IMR 32 cells as seen in wound healing and Boyden chamber invasion assays.

Several studies have shown that withaolides including WA inhibits activation of NF-κB in tumor necrosis factor (TNF)-α stimulated cells by preventing the IκB phosphorylation and degradation [25, 36, 37]. Therefore, we next investigated whether NF-κB pathway is regulated by withanolides in NB cells. Evaluation of the NF-κB activity after 2 μM drug treatment by reporter assay indicated inhibition in activity for all the withanolides when IMR 32 cells were un induced, whereas the fold change in NF-κB activity in terms of untreated control decreased from 26.2 after TNF-α induction to 3.3, 7.1 and 3.9 for WGA, WGB-DA and WGA-TA with a significant *p* value of 0.007, 0.01 and 0.006 respectively (Figure 6A). To determine the mechanism through which withanolides down regulate NF-κB activity, we performed immunoblot and immunofluorescence analysis. IMR 32 cells treated with withanolides showed down regulation of Iκ-Bα in a dose-dependent manner starting at 2 μM. Even though total levels of p65 remained constant, treatment of withanolides inhibited the expression of phospho-p65 (serine 536) starting at 1 μM drug treatment, preventing nuclear localization of NF-κB as shown in (Figure 6B, 6D and 6E). Furthermore, withanolide treatment also inhibited NF-κB downstream genes such as cyclin D1, survivin and N-myc (Figure 6C). Pretreatment of cells with thiol antioxidant NAC, blocked the inhibition of NF-κB with significant *p* values of 0.002, 0.001 and 0.01 for WA, WGB-DA and WGA-TA respectively, indicating thiol oxidation and Hsp90 mediated inhibition of NF-κB by withanolides (data not shown). As NF-kB is known to mediate invasion and metastasis, we performed wound assay as well as Boyden chamber invasion experiment after treatment of IMR 32 cells with withanolides. As expected, we observed migration of cells in the wound area for the control cells which was prevented by treatment of IMR 32 cells with 2 μM of either WGA or WGB-DA or WGA-TA. Analysis of migration of cells through a matrigel further confirmed the observation as treatment of IMR 32 cells with the acetate derivatives WGB-DA and WGA-TA attenuated the migration of more than 80% cells with significant *p* value of 0.01, whereas only 4 μM WGA had blocked the migration and not 2 μM WGA (Figure 6F, 6G and 6H). Overall, the results of the study clearly demonstrate the improved efficacy of the acetate derivatives of withanolides compared to parent compound in modulating key pathways implicated in the pathogenesis of NB and inducing cell death.

### Treatment with WGA-TA causes tumor regression

**Figure 7:**
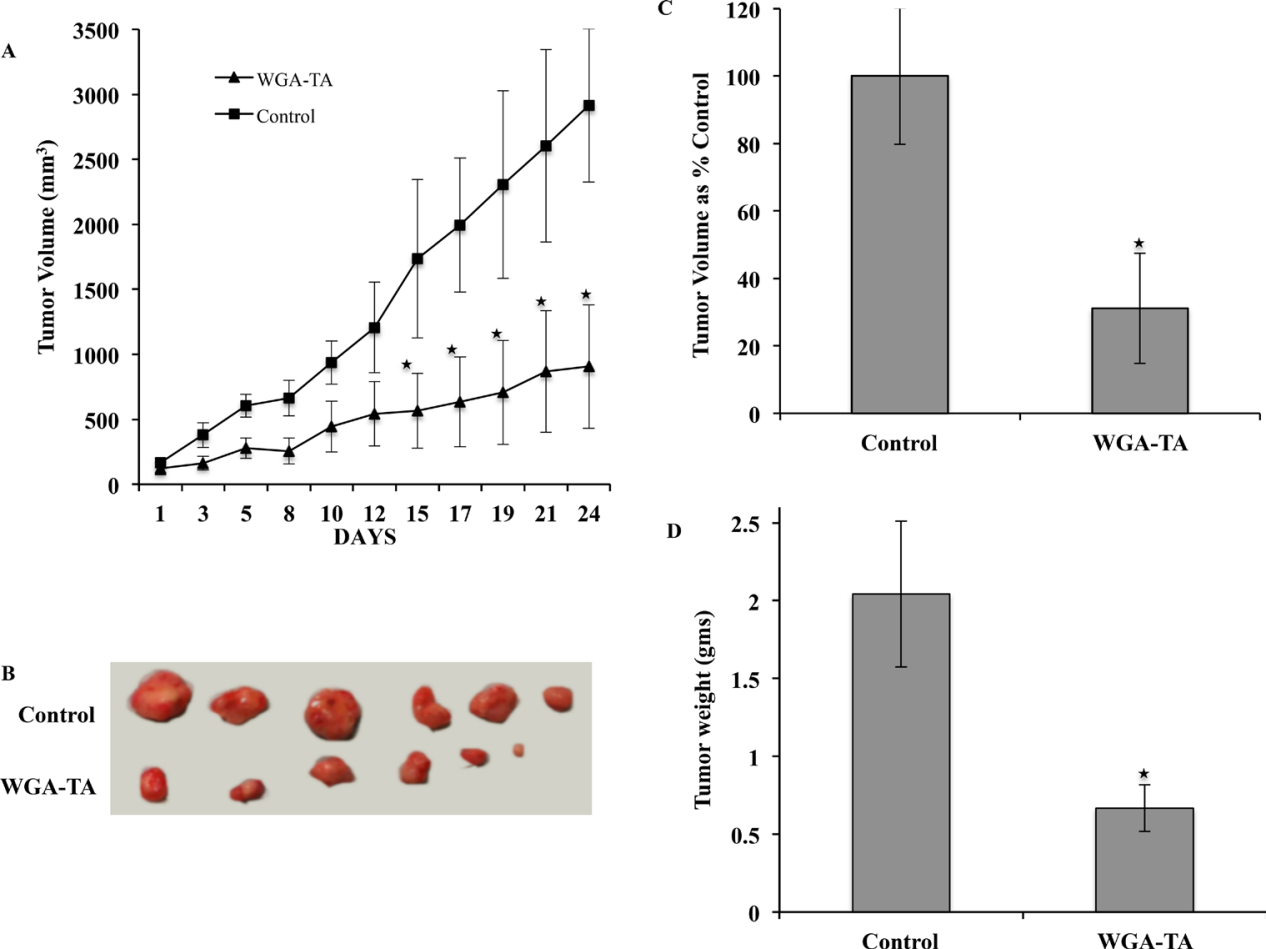
Treatment of IMR32 xenografts with WGA-TA leads to tumor regression (**A** and **B**) Tumor growth curve and explanted tumors. (**C** and **D**) Average tumor volume and tumor weight at the end of the study period of 24 days.

To evaluate the *in vivo* efficacy of the lead compound WGA-TA, in the present study we used human neuroblastoma cell line IMR32 xenografts. Treatment of mice with WGA-TA for 3 weeks resulted in statistically significant decrease in tumor burden starting from day 15 and continued till the end of the study (Figure 7A and 7B). The study was stopped as all the control mice reached the maximum tumor volume approved by our UCUCA protocol. At the end of the study period the withanolide WGA-TA treatment groups showed 68.9% decrease in tumor volume compared to the control groups. In addition, the average tumor weights of the WGA-TA treatment groups decreased to 0.67 gram compared to the control group value of 2.04 gram with a significant p value of 0.01 (Figure 7C and 7D).

## DISCUSSION

NB is an embryonic solid tumor with poor therapeutic outcome for children with high risk tumors. Recent studies have detected activated expression of pAkt and pmTOR in primary neuroblastoma tumors and in differentiated ganglion cells. Additionally, activation of Akt is shown to be associated with poor prognosis in NB [38]. Furthermore, increased expression of several growth factors such as tyrosine receptor kinase B (TrkB), insulin –like growth factor (IGF-1R) and epidermal growth factor (EGFR) that transmit their signals through PI3/Akt/mTOR pathway are known to be associated with drug resistance and increased expression of N-myc in NB [39]. Therefore, targeted therapies using PI3K/Akt/mTOR inhibitors, anti-angiogenic drugs and ALK antagonists are in phase I/II clinical trials either alone or in combination with conventional chemotherapy for refractory or recurrent NB [6]. The problem that will plague these and other single-target drugs is the cancer's ability to activate alternative survival pathways leading to drug resistance and toxicity even in the multi-modality setting. Therefore, novel multi targeted treatment options with fewer adverse effects and toxicity are needed for NB.

Our group over the last few years has demonstrated the anti-cancer properties of novel withanolides from *P.Longifolia* in various cancer models both *in vitro* and *in vivo*. Not only are these natural products extremely safe *in vivo*, but they also function through a unique mechanism of action leading to the simultaneous and selective inhibition of key regulatory pathways including Notch/PI3K/Akt/mTOR/NF-κB in cancers [18, 22, 26, 30]. These natural compounds utilize a multi-targeted approach through their unique mechanism of action: inducing an oxidative stress response (via generation of reactive oxygen species following metabolism of the epoxide on the B-ring of the molecule) in cancer cells coupled with a novel means of inhibiting HSP90 chaperone function through blockade of Cdc37 docking. This in turn prevents kinase activation by HSP90 leading to targeting of key tumorigenic proteins that are implicated in invasion and poor survival of NB. Hence, the present study was designed to investigate the mechanism through which novel natural and semisynthetic withanolides from *P.longifolia* target and induce cell death in NB. The results from the viability assay clearly demonstrated that our novel withanolides are effective in targeting both N-myc amplified and non-amplified cell lines at nano molar IC50 concentrations. In addition, the acetate derivatives WGA-TA and WGB-DA were able to induce potent dose dependent apoptosis and G2/M cell cycle arrest at low concentration of 500 nM compared to 2 μM for withaferin A indicating that they are highly potent in targeting the NB cells compared to the parent withanolide.

Deregulated PI3K/Akt/mTOR pathway proteins and tyrosine kinases are implicated in the development and poor survival of NB. Therefore, to determine how withanolides would modulate these key pathway proteins, we examined their levels of expression by western blot analysis. We observed inhibition of many key survival proteins involved in this pathway like Akt, pAkt, mTOR, p-mTOR and their downstream effectors such as pS6K and p4E-BP1 in both the NB cell lines tested. Further, to elucidate whether withanolides would target MAPK proteins we examined activation of ERK in NB cells. Even though activation of ERK is considered a survival signal, its activation is also associated with oxidative stress induced cell death [40, 41]. Our results revealed prolonged activation of ERK that is associated with proapoptotic properties of ERK until the duration of the experiment (24 h) in NB cell lines, suggesting the possible role of ERK in mediating pro apoptotic signaling after withanolides treatment in NB cells. The observed cleavage of apoptotic markers such as caspase 3 and PARP further confirmed withanolide mediated induction of apoptosis in NB cells. Overall these results, along with the previous studies by Yco et al. [42] that showed inhibition of signal transducer and activator of transcription 3 (STAT3) by WA, indicate that withanolides have the ability to target both the upstream and downstream components of the PI3K/Akt/mTOR/MAPK pathway proteins that are implicated in the poor prognosis of NB. This results in maximal inhibition and/or avoidance of negative feedback loops that are responsible for the development of drug resistance. In addition, targeting multiple nodes of this critical pathway may offer the added benefit of restoring sensitivity to the anti-cancer drugs to which the NB patients have become resistance. Future studies using drug resistant NB cell lines are needed to explore whether our novel withanolides can sensitize the drug resistant NB tumors.

Induction of reactive oxygen species and alteration in redox potential of cancer cells after treatment with chemotherapeutics has been observed in many cancer models as malignant cells are much more susceptible to oxidative stress-induced cell death compared to normal cells [43, 44]. Even though the mechanism through which WA induces ROS is not well defined, studies have shown induction of oxidative stress after WA treatment in breast cancer and glioblastoma [18, 30]. In NB cells we also observed induction of oxidative stress following treatment with withanoldies in a dose dependent manner. Following oxidative stress, heat shock proteins HSP32 and HSP70 were upregulated, while the levels of HSP90 remained the same. WA is known to directly bind to HSP90 and inhibit HSP90 through an ATP independent mechanism leading to degradation of client proteins [27]. Induction of HSP70 and degradation of many client proteins of HSP90 such as Akt, mTOR, N-myc, cyclinD1 clearly indicates inhibition of HSP90 with withanolide treatment in NB cells. To further confirm withanolides bind to HSP90 reactive cysteine, NB cells were pretreated with NAC. Regardless of the mechanism through which NAC pretreatment of NB cells reverses the withanolide effect (either through chemical reaction or through oxidative response), our results revealed inhibition of HSP90, prevention of upregulation of heat shock proteins and attenuation of cell death.

As withanolide treatment of NB cells demonstrated down regulation of Akt, induction of oxidative stress and inhibition of HSP90 function all of which are key regulators of NF-κB, we examined the modulation of NF-κB using reporter assay. Our results demonstrated attenuation of NF-κB in a dose dependent manner due to prevention of nuclear localization of NF-κB as seen by our immunocytochemistry results. Although several withanolides have shown inhibition of NF-κB activation, the exact mechanism through which withanolides inhibit NF-κB is not clearly defined. Previous studies have shown two contributing factors for the suppression of NF-κB. The first is the prevention of Iκ-Bα phosphorylation and degradation with subsequent reduction of nuclear NF-κB and the second mechanism is through targeting IKKs due to HSP90 inhibition. Results from our immunoblot analysis revealed degradataion of Iκ-Bα and a decrease in the phosphorylation of p65 at ser536 in a dose-dependent manner without altering the total levels of p65. Studies by Paimela et al. have shown modulation of NF-κB activity and down regulation of p65 at serine 536 via HSP70 regulation after celastrol treatment of retinal pigment epithelial cells [45]. Since we observed up regulation of HSP70 due to induction of oxidative stress and inhibition of HSP90 activity, one possible mechanism through which withanolides inhibit NF-kB activation in NB cells may be via suppression of nuclear translocation of NF-κB through attenuation of serine 536 phosphorylation of p65. Further analysis of the target genes of NF-κB revealed down regulation of cell cycle regulator cyclinD1, survivin and N-myc. Given that in majority of high-risk NBs cell cycle exit and terminal differentiation that occurs during neuroblast development are disrupted by high N-myc expression. Additionally, Akt phosphorylation is known to be correlated with N-myc and PI3K/Akt signaling is known to prevent degradation of N-myc. Hence, suppression of N-myc, which is a key prognostic factor in high-risk NB, is targeted by withanolides through modulation of multiple factors such as Akt, mTOR, GSK-3β, NF-κB etc. Many of the genes that are involved in the migration and metastasis of cancer cells like vascular endothelial growth factor and matric metallo proteinases are regulated by NF-κB. Therefore, we evaluated whether suppression of NF-κB by withanolides can prevent migration of NB cells using wound healing assay and matrigel invasion assay. Our results clearly demonstrated that our novel withanoldies prevents migration and invasion of NB cells through down regulating NF-κB.

In summary, this study for the first time reveals the detailed mechanism through which natural withanolide, WGA isolated from *P.longifolia,* and its semi synthetic acetate derivatives (WGB-DA and WGA-TA) induce cell death in NB cells. Based on the results from our study, one possible mechanism through which withanolides prevent migration and potentiate apoptosis and cell cycle arrest in NB cells is through induction of oxidative stress coupled with a novel mechanism of HSP90 inhibition, which in NB targets key tumorigenic proteins such as Akt, mTOR, NF-κB and N-myc. Down regulation of these oncogenic signal transduction pathway proteins in turn leads to suppression of migration and invasion of NB cells. Convincing evidence from our *in vitro* results and given that naturally derived withanolides unlike chemotherapeutics are less toxic, support further translational development of withanolides as a novel anticancer drug for NB patients. However, future *in vivo* efficacy and toxicity studies in NB xenografts are warranted to validate the clinical application of our novel withanolides as potential therapeutics for NB.

## MATERIALS AND METHODS

### Cell culture and reagents

Validated human NB cell lines IMR 32, SH-EP1, SK-N-As and GOTO were grown in Minimum Essential Medium (MEM; Life Technologies, Grand Island, NY) supplemented with 10% fetal bovine serum (FBS; Sigma-Aldrich, St. Louis, MO) and 1% penicillin/streptomycin (Life Technologies, Grand Island, NY). IMR 32 medium is additionally supplemented with 1% MEM non-essential amino acids (100X; Sigma-Aldrich), 2 mM of L-glutamine (200 mM; Sigma-Aldrich), *1 mM sodium pyruvate (100mM; Sigma-Aldrich), and 1500 mg/L of sodium bicarbonate (7.5%; Sigma-Aldrich).* All the cell lines were incubated in a humidified atmosphere of 5% CO_2_ in air at 37°C. The structure of the withanolides used in the study namely WA, WGA, WGA-TA and WGB-DA were previously described [15] and were obtained from Dr. Barbara Timmermann's laboratory (Lawrence, Kansas). Reagents used in the flow cytometry analysis for cell cycle and apoptosis such as propidium idodide (PI), RNase were obtained from Sigma-Aldrich ((St. Louis, MO) and Annexin V-FITC was obtained from BD biosciences (San Diego, CA). N-acetyl-L-cysteine (NAC) was obtained from Sigma-Aldrich (St. Louis, MO) and CM-H_2_DCFDA was from Molecular Probes (Grand Island, NY).

### Evaluation of cytotoxicity

To measure the drug efficacy of withanolides on NB cell lines IMR 32, SH-EP1, SK-N-As, and GOTO cells were seeded in a 96 well plates at a density of 3000 cells per well and the viability was determined by the colorimetric CellTiter96 Aqueous MTS assay as per the manufacturer's protocol (Promega, Fitchburg, WI). The cells were treated with serial dilutions ranging from 0.0195–10 μM of WA, WGA-TA or WGB-DA and incubated for either 24 h or 72 h at 37°C in a CO_2_ humidified chamber. At the end of the treatment period, absorbance at 490 nm was recorded on a BioTek Synergy 2 plate reader (BioTek, Winooski, VT) after the addition of MTS reagent and cytotoxicity was expressed as a percentage of control untreated cells. For NAC co-treatment with withanolides, CellTiter-Glo luminescent assay (Promega, Fitchburg, WI) was used to determine the viability to avoid the auto-reductive potential of NAC that interferes with MTS. The viability was determined by measuring the adenosine triphosphate levels (ATP) as per the manufacturer's protocol. Experiments were performed in triplicate and the values were expressed as means ± S.D. The half-maximal inhibitory concentrations (IC50) and *p* values were calculated using Graphpad Prism and excel.

### Flow cytometry for the analysis of cell cycle and apoptosis

The NB cell lines IMR 32 and GOTO grown to 60–80% confluence in 60 mm plates were treated with 0.25–2 μM of WA, WGA-TA or WGB-DA for 24 h. The cells were then harvested, resuspended in 0.43 mL of ice cold 1X phosphate buffered saline (PBS) followed by 1 mL of ice cold ethanol to get a 70% ethanol fixative solution and stored at −20°C until analysis. Finally, the cells were pelleted by centrifugation, stained with propidium iodide (PI) solution (40 μg/mL PI and 100 mg/mL RNaseA), and incubated at 37°C for 30 minutes before cell cycle analysis using CyAn™ ADP Analyzer (Beckman Coulter, Inc., Brea, CA) at the University of Michigan Flow Cytometry Core. Each experiment was repeated thrice and only viable cells without DNA fragmentation were analyzed using FlowJo software (Tree Star, Inc., Ashland, OR).

Phosphatidylserine staining with annexin on the outer leaflet of the cell membranes for apoptotic cells, and DNA staining by PI for necrotic and late apoptotic cells was performed for the analysis of cell death. IMR 32 and GOTO cells grown and treated with varying concentrations of WA or WGA-TA or WGB-DA for 24 h as in cell cycle analysis were trypsinized, collected and washed once with Annexin binding buffer [18]. The cells were then stained using Annexin V- fluorescein isothiocyanate (FITC)/PI staining according to the manufacturer's protocol (BD Biosciences, San Diego, CA) and induction of apoptosis was measured using the CyAn™ ADP Analyzer. Experiments were repeated thrice to confirm the results.

### Immunoblot analysis

The NB cell lines IMR 32 and GOTO grown to 50% confluence was treated with varying concentrations of withanolide derivatives WA, WGA-TA or WGB-DA for 24 h. For NAC treatment the IMR 32 cells were pretreated for 1 h with 5 mM NAC before drug treatment. The cells were then collected in lysis buffer and the proteins were quantified using the BCA Protein Assay (Thermo Scientific, Rockford, IL) as previously described [32]. Equal amounts of proteins were separated using SDS-PAGE and then transferred onto a Hybond nitrocellulose membrane (GE Healthcare Life Sciences, Piscataway, NJ). The membranes were blocked and probed with appropriate dilutions of the primary and secondary antibodies then visualized using Super signal Chemiluminescence Reagent (Thermo Scientific, Rockford, IL) as previously described [18]. Actin control was used to ensure equal loading of proteins and for transfer efficiency. Densitometric analysis was performed using Image J software (NIH). The cytosolic and nuclear fractions were prepared after treatment of IMR32 cells with withanolides using NE-PER nuclear and cytoplasmic extraction kit (Thermo Scientific, Rockford, IL) as per the manufacturer's protocol. The integrity of the cytosolic and nuclear fractions were verified by immunoblotting for tubulin and lamin A/C.

### Measurement of reactive oxygen species (ROS)

A general oxidative stress indicator CM-H_2_DCFDA that fluoresces upon oxidation was used to determine the accumulation of intracellular peroxide type reactive oxygen species. IMR 32 cells were incubated at 37°C with 20 μM CM-H_2_DCFDA in 1X PBS for 1 h for preloading of the probe and then plated in 96 well plates at a density of 20,000 cells per well in phenol red free medium. After 30 min, varying concentrations of withanolides (1–5 μM) and/or 5 mM NAC was added and the fluorescence was assessed 3–5 h post treatment using BioTek Synergy plate reader with excitation and emission filters of 485 nm and 528 nm respectively.

### Immunocytochemistry

Approximately 25,000 IMR 32 cells were seeded in an eight well chamber slides (Nalgene Nunc International, Penfield, NY) and were allowed to attach overnight. The cells were treated with 2 μM of WA, WGA-TA or WGB-DA for 24 h and then fixed using 4% paraformaldehyde for 30 min. The cells were washed three times with 1X phosphate buffered saline (PBS), permeabilized using 0.3% triton X-100 for 15 min. after which they were blocked with 5% normal goat serum (Cell Signaling Technology) for 1 h at room temperature. The primary antibody for p65 (cell Signaling Technology) at a dilution of 1:500 in 1X PBS was added to the cells and the cells were incubated at 4°C overnight. After incubation with primary antibody, the cells were washed with 1X PBS three times and then incubated with secondary antibody (1:1000; anti-rabbit IgG Fab2 Alexa Fluor 555, Cell Signaling #4413) for 1 h at room temperature. Finally the cells were washed thrice with 1X PBS, mounted on a slide with ProLong Gold Antifade with DAPI (Cell Signaling Technology) and imaged using NIS Elements software and Nikon A1Rsi Confocal Microscope.

### NF-κB reporter assay

The NB cell line IMR 32 grown to 60–70% confluence was transfected with NF-κB reporter plasmid pBVIx-Luc [46] along with Renilla luciferase plasmid (a gift from Dr. Eric R. Fearon, University of Michigan) by lipofectamine 2000 as per the manufacturer's protocol. 24 h post transfection, the cells were treated with either with 2 μM WA, WGA-TA or WGB-DA for 3 h before treatment with TNF-α for another 16 h. NF-κB activity was then measured using Dual-Luciferase Reporter (DLR) assay kit from Promega as per the manufacturer's instruction. For NAC treatment the cells were pretreated with 5 mM NAC 1 h before drug treatment.

### Scratch wound healing assay

IMR 32 cells grown to 70–80% confluence as a monolayer were scratched using a pipette tip to create a wound. The unattached cells were washed with fresh medium and then the cells were treated with 2 μM of each withanolides. The cells were washed with 1X PBS and then photographed using a phase contrast microscope at time 0 h (immediately after the scratch) and 24 h after scratch to monitor the migration of cells into the wounded area [47].

### Matrigel invasion assay

Assays were done in triplicate in inversion chambers pre-coated with Matrigel (BD Biosciences) to assess the invasive properties of the cells. The inserts were transferred in to the 24-well plate containing 0.75 mL culture medium with 10% FBS and approximately 50,000 IMR 32 cells in 0.5 mL of serum free medium were seeded on to the atypical side of the insert. The plates were incubated at 37°C, 5% CO2 atmosphere for 24 h after treatment with 2 μM withanolides (WA, WGA-TA or WGB-DA). At the end of the treatment the non-invaded cells were removed using a cotton swab and the invaded cells at the lower side of the insert membrane was stained with crystal violet, air dried and imaged. For calorimetric estimation of the invaded cells, the inserts were treated with 150 μL of 10% acetic acid and the absorbance was measured at 560 nm using a BioTek plate reader.

### IMR32 xenografts

Athymic nude mice were injected with approximately 3million IMR32 human neuroblastoma cell lines subcutaneously in the right flank. Once the tumors reached the size of 4mm^2^ diameter the mice were randomized into control and WGA-TA treatment groups. Control groups received solvent where as the treatment groups received 6mg/kg WGA-TA in 50mg/ml captisol. Tumor size and volume were measured thrice weekly and the mice were sacrificed once the tumor volume reached greater than 2000 mm^3^. All procedures were performed in accordance with the University of Michigan Animal care and use committee (UCUCA) approved protocol.

### Statistical analysis

The IC_50_ curves were generated using best fit nonlinear sigmoidal dose response curves in GraphPad Prism 6. Differences between mean values were determined by Student's unpaired *t*-test using Excel or GraphPad Prism. Each experiment was repeated at least three times and data are presented as mean values with standard deviation/ standard error as error bars. *P*-values less than 0.05 were set as significance for all the calculations.
